# Proteomic Profiling of the Outer Membrane Fraction of the Obligate Intracellular Bacterial Pathogen *Ehrlichia ruminantium*

**DOI:** 10.1371/journal.pone.0116758

**Published:** 2015-02-24

**Authors:** Amal Moumène, Isabel Marcelino, Miguel Ventosa, Olivier Gros, Thierry Lefrançois, Nathalie Vachiéry, Damien F. Meyer, Ana V. Coelho

**Affiliations:** 1 CIRAD, UMR CMAEE, Site de Duclos, Prise d’eau, F-97170, Petit-Bourg, Guadeloupe, France; 2 INRA, UMR1309 CMAEE, F-34398, Montpellier, France; 3 Université des Antilles et de la Guyane, 97159, Pointe-à-Pitre cedex, Guadeloupe, France; 4 Instituto de Biologia Experimental e Tecnológica, Apartado 12, 2780-901, Oeiras, Portugal; 5 Instituto de Tecnologia Química e Biológica António Xavier, Universidade Nova de Lisboa, Av. da República, 2780-157, Oeiras, Portugal; 6 Université des Antilles et de la Guyane, Institut de Biologie Paris Seine, UMR7138 UPMC-CNRS, Equipe Biologie de la Mangrove, UFR des Sciences Exactes et Naturelles, Département de Biologie, BP 592, 97159, Pointe-à-Pitre cedex, Guadeloupe, France; University of Minnesota, UNITED STATES

## Abstract

The outer membrane proteins (OMPs) of Gram-negative bacteria play a crucial role in virulence and pathogenesis. Identification of these proteins represents an important goal for bacterial proteomics, because it aids in vaccine development. Here, we have developed such an approach for *Ehrlichia ruminantium*, the obligate intracellular bacterium that causes heartwater. A preliminary whole proteome analysis of elementary bodies, the extracellular infectious form of the bacterium, had been performed previously, but information is limited about OMPs in this organism and about their role in the protective immune response. Identification of OMPs is also essential for understanding *Ehrlichia*’s OM architecture, and how the bacterium interacts with the host cell environment. First, we developed an OMP extraction method using the ionic detergent sarkosyl, which enriched the OM fraction. Second, proteins were separated via one-dimensional electrophoresis, and digested peptides were analyzed via nano-liquid chromatographic separation coupled with mass spectrometry (LC-MALDI-TOF/TOF). Of 46 unique proteins identified in the OM fraction, 18 (39%) were OMPs, including 8 proteins involved in cell structure and biogenesis, 4 in transport/virulence, 1 porin, and 5 proteins of unknown function. These experimental data were compared to the predicted subcellular localization of the entire *E. ruminantium* proteome, using three different algorithms. This work represents the most complete proteome characterization of the OM fraction in *Ehrlichia spp*. The study indicates that suitable subcellular fractionation experiments combined with straightforward computational analysis approaches are powerful for determining the predominant subcellular localization of the experimentally observed proteins. We identified proteins potentially involved in *E. ruminantium* pathogenesis, which are good novel targets for candidate vaccines. Thus, combining bioinformatics and proteomics, we discovered new OMPs for *E. ruminantium* that are valuable data for those investigating new vaccines against this organism. In summary, we provide both pioneering data and novel insights into the pathogenesis of this obligate intracellular bacterium.

## Introduction

The *Rickettsiales Ehrlichia ruminantium* is an obligate intracellular bacterium that causes heartwater, a fatal tick-borne disease of ruminants, which is found in the islands of the Indian Ocean and the Caribbean, and in Africa [1]. *E*. *ruminantium* is transmitted by *Amblyomma* ticks and infects the endothelium of blood vessels. It has a complex life cycle with two distinct developmental forms found within mammalian host cells [2]. Initially, the infectious forms of the bacterium (elementary bodies, or EBs) adhere to host target cells and are internalized. Then, inside of intracytoplasmic vacuoles, they differentiate into a replicative, non-infectious form, the reticulate body (RB). After 5 to 6 days of intracellular multiplication, disruption of host cells leads to the release of numerous infectious EBs, initiating a new infectious cycle [1,3].

Current control methods for heartwater consist of a combination of vector control, using acaricides, and immunization against *E*. *ruminantium*. Different types of vaccines (inactivated, attenuated, recombinant) are currently being tested experimentally, but they have displayed limited efficacy, thus far, due to the genetic and antigenic diversity of *E*. *ruminantium* strains [3–8]. At this time, the only commercially available vaccine is based on the administration of infected blood to ruminants, followed by treatment with antibiotics; however, this remains an expensive, high-risk method [3].

Many studies of Gram-negative bacteria, such as *Legionella pneumophila*, *Bartonella henselae*, *Pseudomonas syringae*, *Campylobacter jejuni*, and *Mannheimia haemolytica*, have focused on outer membrane proteins (OMPs), because they have proven to be good targets for vaccine development [9–13]. Indeed, the OM of such pathogens represents an important dynamic interface between the bacterium and its environment. It serves as a selective barrier controlling the passage of nutrients and waste products into and out of the cell, and it also creates a chemically distinct periplasmic compartment, where important processes, such as the degradation of harmful substances from the environment or certain types of respiration, can occur [14,15]. OMPs are involved in the integrity and stability of the bacterial envelope, passive and active transport of substrates and nutrients, cell-to-cell communication, adhesion to host cells, and virulence [16].

Prospective proteomic analysis of *E*. *ruminantium*, cultivated in host endothelial cells, has already provided information about OMPs that are potentially implicated in bacterial infection and survival, such as members of the major antigenic protein (*map)* gene cluster [17,18]. Despite significant evidence implicating this gene family in immune protection in *Ehrlichia* and *Anaplasma* [19,20] and even strain penetrance in *Anaplasma* [21], our understanding of the biological role of this gene family is incomplete. However, studies on the differential expression of genes encoding OMPs has permitted us to understand the adaptation of these bacteria to the environment inside their vector, the tick, and to transmission to the mammalian host [22,23].

The aim of this study was to characterize the proteome of the OM fraction from infectious *E*. *ruminantium* EBs. To obtain an enriched OM fraction, we optimized a sarkosyl-based enrichment protocol that selectively solubilizes the inner and cytoplasmic membranes of Gram-negative bacteria, with no effect on the OM subcellular fraction [24]. We identified 46 unique proteins in the OM fraction using one-dimensional gel electrophoresis coupled with liquid chromatography-mass spectrometry (1DE-nanoLC-MALDI-TOF/TOF). Of these, 18 were known or predicted prototypical OMPs, while the others were of inner membrane (n = 5) or cytoplasmic (n = 23) origin or were chaperones. We compared our experimental results to the total set of *E*. *ruminantium* OMPs by combining results from three subcellular localization prediction algorithms and 34% of the total OMPs predicted from the genome were detected in the obtained OM fraction. We concluded that our method enriched OMPs. These results provide a better understanding of *Ehrlichia* OM architecture and may lead to the identification of potential vaccine candidates.

## Importance

*Ehrlichiae* are obligate intracellular bacteria with a unique developmental cycle that includes attaching to and entering eukaryotic host cells, a process mediated by proteins in their outer membrane (OM). Thus far, few experimental data on ehrlichial OM proteins are available. To gain insight into the protein composition of the ehrlichial OM, we performed proteome analysis on OM fractions from *Ehrlichia ruminantium* elementary bodies, the infectious form of this bacterium. We compared our experimental results with an *in silico* analysis of the *E*. *ruminantium* proteome. We identified 18 proteins, whose OM localization was supported by both studies, and were, therefore, very likely to be located in the *E*. *ruminantium* OM. Among these proteins, 6 are completely new discovered OMPs and are therefore of importance as potential vaccine antigens. These results provide the first comprehensive overview of OM proteins in an *Ehrlichia* species and pave the way for developing novel therapeutic strategies to disrupt the OM or processes essential for its function

## Materials and Methods

### *Ehrlichia ruminantium* cultivation

*E*. *ruminantium* strain Gardel (from Guadeloupe, FWI) was routinely propagated in bovine aorta endothelial cells (BAE) as previously described [25]. One-hundred and twenty hours post-infection, when cell lysis occurs, infectious EBs were harvested and purified using a multistep, 20,000 × *g* centrifugation protocol, as described elsewhere [26,27]. Purified EBs were stored at -80°C in sucrose-phosphate-glutamate (SPG) buffer, pH 7.4.

### Preparation of the OM fraction from *E*. *ruminantium* EBs

**Fig 1:**
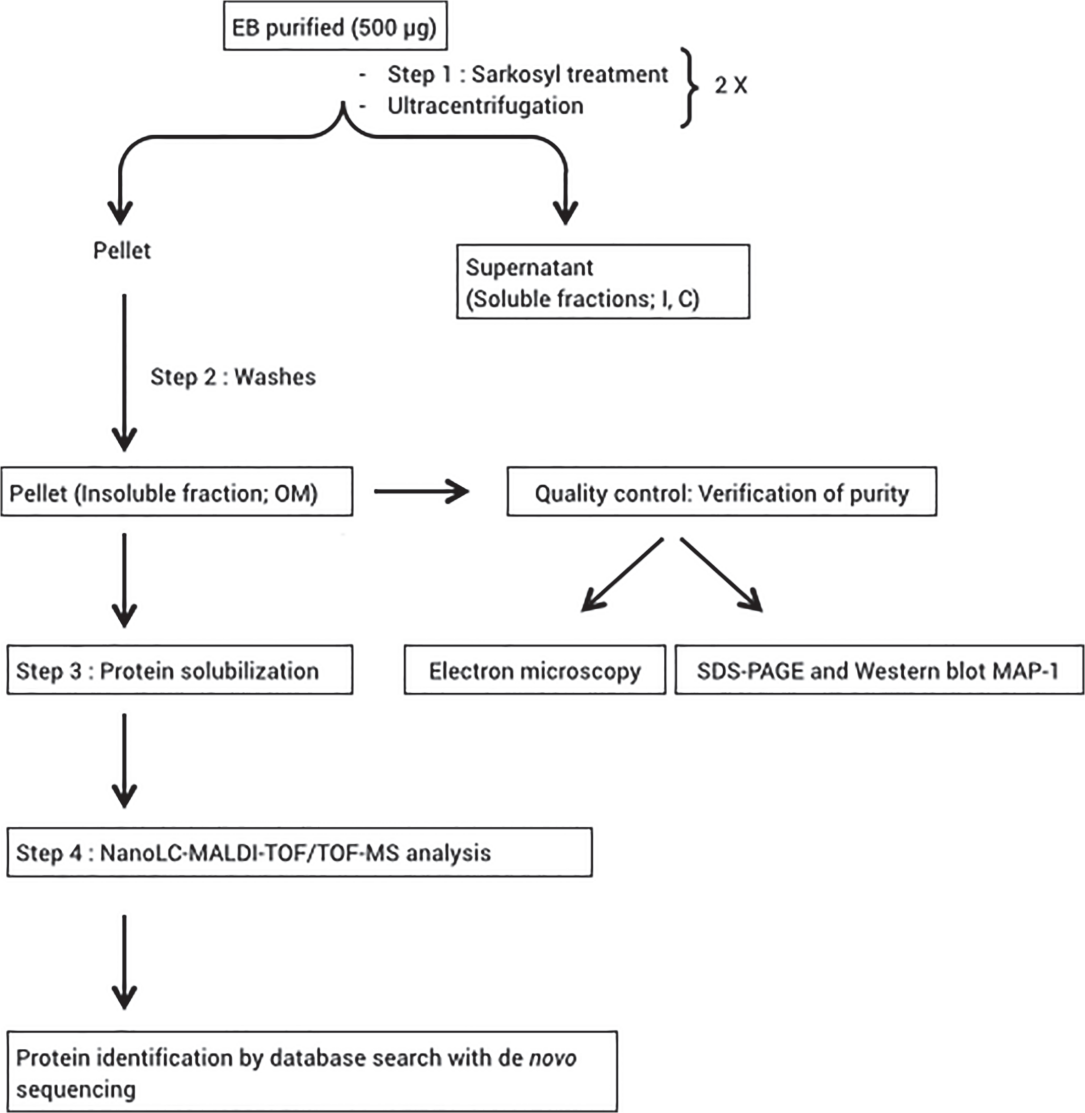
Experimental workflow for *E*. *ruminantium* subcellular fractionation and proteome characterization. **OM**, outer membrane; **I**, inner membrane; **C**, cytoplasm.

Subcellular fractionation was performed as described by Ohashi *et al*. [28], modified as follows. Purified EBs stored in SPG were washed in phosphate-buffered saline (PBS, pH 7.4) with a protease inhibitor cocktail (Roche), at 20,000 × *g* for 30 min at 4°C. Protein content was measured with the microBCA quantification kit (Sigma), according to the manufacturer’s instructions. Five hundred micrograms EBs were pelleted and resuspended in PBS containing 0.1% (v:v) sodium N-laurosyl sarcosine (sarkosyl; Sigma), DNAse (50 μg/mL), RNAse (50 μg/mL), MgCl_2_ (2.5 mM), and protease inhibitors (Roche), and then incubated for 30 min at 37°C. The sarkosyl treatment was repeated twice, followed by ultracentrifugation at 20,000 × *g* for 30 min at 4°C (Fig. 1). After the first separation, the insoluble pellet containing the OM fraction was washed twice in PBS and centrifuged at 20,000 × *g* for 30 min at 4°C to remove residual detergent (Step 2); the final pellet was resuspended in PBS containing protease inhibitors, and then stored at 4°C. Total protein concentration was determined using the 2D Quant Kit (GE Healthcare). Independent biological triplicates were carried out for OMP characterization (Fig. 1).

### Evaluation of OM enrichment protocol

1. **Transmission Electron Microscopy (TEM)**. Samples were pre-fixed at 4°C in 2.5% (v/v) glutaraldehyde in PBS (pH 7.2). After a brief rinse with 1 × PBS, samples (intact EBs or OM complex) were fixed for 45 min at 25°C in 1% (w/v) osmium tetroxide in the same buffer, rinsed in distilled water and post-fixed with 2% (w/v) aqueous uranyl acetate for 1 h at 25°C before being embedded in epoxy resin. Two grids containing 4–5 ultrathin sections (60 nm thick) were observed using a Tecnai G2 TEM at 200 kV [29]. The TEM micrographs presented in this study are representative of all samples.
2. **SDS-PAGE and Western blots to monitor OM fraction**. Biological samples (15 μg) were precipitated in acetone for 3 h at -20°C and centrifuged at 20,000 × *g* for 10 min at 4°C. The pellet was solubilized in NuPAGE LDS Sample Buffer loaded on NuPAGE Novex 4–12% Bis-Tris polyacrylamide gels, and electrophoresis was carried out for 40 min at 200 V. Proteins were transferred to polyvinylidene difluoride (PVDF) membranes (Millipore, USA). The membranes were blocked for 1 h in PBS with 0.05% (v/v) Tween 20 and 5% (w/v) milk, and then incubated with anti-MAP1 mouse monoclonal antibody (mAB) (4F10B4, Abcam) at a dilution of 1:2,000 for 1 h. Anti-Map1 monoclonal antibody was used as a specific OM marker. Membranes were washed three times in PBS with 0.05% (v/v) Tween 20 for 10 min, followed by incubation with the appropriate phosphatase alkaline-conjugated secondary antibodies (Sigma) at a 1:2,000 dilution for 1 h. Finally, membranes were developed using 5-bromo-4-chloro-3’-indolyphosphate/nitro-blue tetrazolium (BCIP/NBT) substrate (Roche) [17].

### Proteome Characterization

1. **1D gel electrophoresis for proteomics analysis**. Forty μg intact EBs or OM fraction (from ERGp45, p52, and p57) were precipitated in acetone for 3 h at -20°C and centrifuged at 20,000 × *g* for 10 min at 4°C. Pellets were resuspended in 5 μL solubilization buffer [7 M urea, 2 M thiourea, 4% (w/v) 3-[(3-cholamidopropyl) dimethylammonio]-1-propanesulfonate (CHAPS), and 30 mM Tris; Step 3 in Fig. 1]. After protein solubilization, 6 μL loading buffer [0.5 M dithiothreitol (DTT), 10% (w/v) sodium dodecyl sulfate (SDS), 250 mM Tris, 30% (v/v) glycerol, and 0.02% (w/v) bromophenol blue] was added. Samples were vortexed, and 9 μL water was added followed by agitation overnight at room temperature. Finally, samples were centrifuged at 16,000 × *g* for 2 min, and supernatants were loaded on NuPAGE Novex 4–12% Bis-Tris polycacrylamide gels; electrophoresis was performed for 40 min at 200 V. Gels were stained for 24 h using colloidal Coomassie Blue, and then washed 3 times in double distilled water [17].
2. **In-gel digestion**. For the evaluation of the optimized protocol to obtain an OMP enriched fraction, the more intense gel bands were excised. Previously to the NanoLC-MALDI-TOF/TOF analysis and in order to extend the number of proteins identified starting from simpler peptide digests, the OMP enriched fraction was separated by SDS-PAGE and each gel lanes was sliced. For in-gel digestion each band or slice was cut into 1 mm^3^ gel pieces, and Coomassie Blue was washed off with alternating water and 50% (v/v) acetonitrile (ACN) treatments until the gel pieces were transparent. Proteins were in-gel reduced with 10 mM dithiothreitol (DTT), alkylated with 55 mM iodoacetamide. Next, 6.7 ng/μL modified porcine trypsin (Promega) in 50 mM NH_4_CO_3_ was added to each gel band/slice. Digestion was performed at 37°C overnight. Peptides were extracted from the gel by washing it with 5% (v/v) formic acid, followed by two ACN washes. Digestion supernatants and extracted peptides were added, dried in a SpeedVac concentrator, and reconstituted in 5% (v/v) formic acid [30].
3. **NanoLC-MALDI-TOF/TOF analysis**. Chromatographic peptide separation was performed on a Thermo EASY-nLC 1000 with a pre-column Acclaim PepMap 100 C18 (75 μm × 2 cm) used as the Peptrap and an Acclaim PepMap RSLC C18 (50 μm × 15 cm) as the chromatographic separation column (Step 4, Fig. 1). A chromatographic gradient was established using mixed volumes of 0.1% (v/v) formic acid in water (buffer A) and 0.1% (v/v) formic acid in acetonitrile (buffer B, all LC-MS grade, from MERCK); peptides were eluted at a constant rate of 2 mL/min for 40 min in 5–40% (v/v) buffer A, according to their hydrophilic/hydrophobic properties. Peptide fractions were spotted onto MALDI plates and co-crystalized with 5 mg/mL alpha-cyano-4-hydroxycinnamic acid using a Micro-Spotter (Sunchrom). Peptide mass spectra were acquired with an Applied Biosystems 4800 Plus MALDI TOF/TOF Analyzer apparatus in both MS and MS/MS mode. Positively charged ions were analyzed in the reflectron mode over an m/z range of 800–3,500 Da. Each MS spectrum was obtained in result-independent acquisition mode with a total of 800 laser shots per spectra and a fixed laser intensity of 3,500 V. Calibration was performed using Des-Arg-bradykinin (904.468 Da), angiotensin 1 (1,296.685 Da), Glu-Fibrinopeptide B (1,570.677 Da), ACTH (1–17 clip) (2,093.087 Da), and ACTH (18–39 clip) (2,465.199 Da) (Calibration Mix from Applied Biosystems). Fifteen s/n best precursors from each MS spectrum were selected for MS/MS analysis. MS/MS analyses were performed using collision-induced dissociation (CID) assisted with air, using collision energy of 1 kV and a gas pressure of 10^6^ Torr. Two thousand laser shots were collected for each MS/MS spectrum using a fixed laser intensity of 4,500 V. Raw data were generated using 4000 Series Explorer Software v3.0 RC1 (Applied Biosystems, Foster City, CA, USA), and all contaminant m/z peaks originating from human keratin, trypsin autodigestion, or matrix were placed on the exclusion list used to generate the peptide mass list used in the database search [17].
4. **Database query**. To identify proteins, Mascot generic format files combining MS and MS/MS spectra were used to interrogate a non-redundant protein database using a local Mascot v2.2 license from Matrix Science and the Global Protein Server (GPS) v3.6 (Applied Biosystems). Search parameters for the MS/MS spectra were as follows: i) the Uniprot (2013) sequence database (*E*. *ruminantium* with isoforms) was used; ii) taxonomy was set to “all entries” (302,409); iii) variable modifications were considered [i.e., carbamidomethylation (Cys), deamidation (Asn and Gln), and oxidation (Met, Pro, Lys, Arg)]; iv) two missed cleavage sites were allowed; v) precursor tolerance was set to 50 ppm and MS/MS fragment tolerance to 0.5 Da; vi) peptide charge was 1+; and vii) the algorithm used trypsin as the enzyme. A protein candidate provided by this MS/MS search was considered valid if the global Mascot score was >40 at a significance level of p<0.05, if at least one peptide was identified with 95% confidence, and if it was found in at least two of the three biological replicates.

### *In silico* genome analysis

The publicly available proteome of the *E*. *ruminantium* strain Gardel, which was extracted from the Uniprot database [31] in FASTA format, was used for bioinformatics studies. The subcellular localization of the 948 *E*. *ruminantium* protein-coding genes was predicted using three global programs: PSORTb 3.0 [32], CELLO 2.5 [33], and MetaLocGramN [34]. The predicted utilization locations of each protein were filtered from raw software output using in-house scripts written in the R programming language and exported to Excel. In some cases, CELLO 2.5 predicted multiple localization sites for the same protein. The proteins involved were grouped under the heading “unknown localization.”

As a result of the varying predictions for a given protein, the consensus prediction was calculated using a majority vote procedure. If two of three algorithms agreed on localization, this localization was attributed to the protein. As for the remaining results, when outer or inner membrane localization was predicted by only one program, protein subcellular localization was refined manually, based on the experimental data in the literature, or the presence of signal peptides, transmembrane domains using dedicated algorithms (Table 1; S1 Table).

**Table 1:** Subcellular localization of *E*. *ruminantium* strain Gardel proteins as predicted by PSORTb 3.0, CELLO 2.5, MetaLocGramN, and consensus.

Subcellular localization	PSORTb 3.0		CELLO 2.5		MetaLocGramN		Consensus prediction	
	Number	%	Number	%	Number	%	Number	%
Cytoplasmic	490	51.6	461	48.6	526	55.4	499	52.6
Periplasmic	4	0.4	9	0.9	1	0.1	1	0.1
Inner Membrane	198	20.8	109	11.4	192	20.2	124	13.0
Extracellular	9	0.9	23	2.4	158	16.6	16	1.6
Outer membrane	11	1.1	90	9.4	71	7.4	52	5.4
Unknown	236	24.8	256	27.0	0	0	256	27.0
Total	948		948		948		948	

Percentages correspond to the number of proteins in each compartment relative to the total number of proteins.

## Results

### Enrichment of *E*. *ruminantium* OM fraction

**Fig 2:**
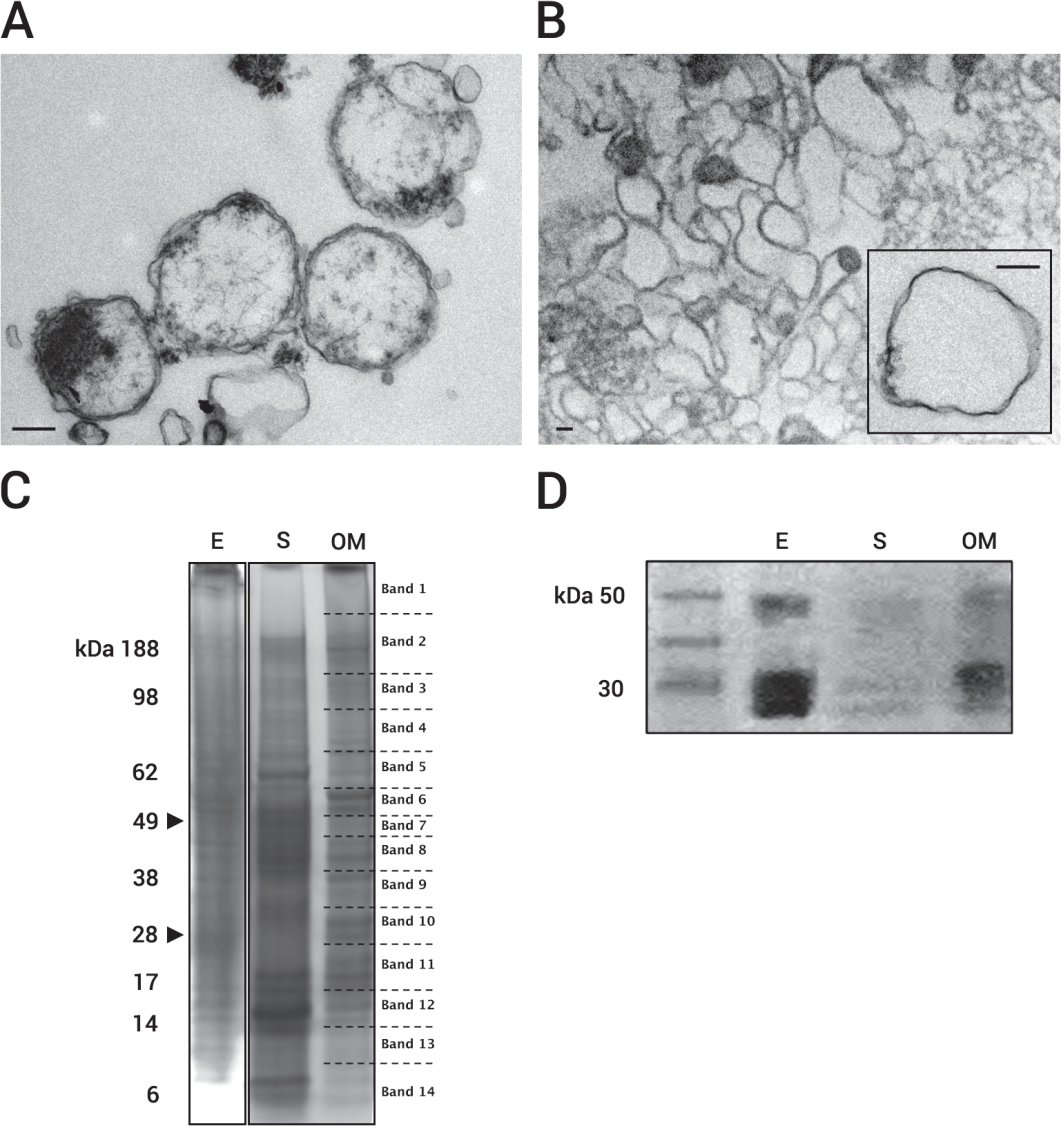
Evaluation of OM isolation quality. Transmission electron microscopy of (A) purified *E*. *ruminantium* and (B) the insoluble precipitate after 0.1% sarkosyl treatment; scale bar = 200 nm. (C) SDS-PAGE and (D) Western blot of **E** (elementary bodies), **S** (sarkosyl-soluble fraction), and **OM** (outer membrane fraction) using monoclonal antibodies against Map1. **Band 1**: Map1-14, X5HG56, GroEL; **Band 2**: Map1+1, Map1, Map1-6, VirB10, VirB4, GroEL, PyrE, Q5HAR6, X5HG56, 30S-S8; **Band 3**: Map1+1, Map1-6, Map2, GroEL, PyrE, Q5HAR6, X5HG56, Q5FGC2, Q5HBI2, Q5FHJ9; **Band 4**: Map1, Map1-6, VirB4, GroEL, DnaK, BamA, FusA, Pnp, Q5HAR6, X5HG56, Q5FH07, Q5HBS6; **Band 5**: VirB4, VirB10, VirB11, DnaK, HtpG, GroEL, FusA, 30S-S1, Q5FGV5, Q93FS2; **Band 6**: Map1, Map1-14, VirB10, PleD, GroEL, DnaK, FtsZ, 30S-S1, Q5HB83, Q5FGA7, Q5HBE1; **Band 7**: Map1-14, GroEL, DnaK, FtsZ, HtpG; **Band 8**: Map1-6, Map1, GroEL, DnaK, FtsZ, BamA; **Band 9**: Map1-6, Map1, Map1+1, Map1-14, GroEL, DnaK, BamA, Q5FFE6, Q5HAR6; **Band 10**: Map1-11, Map1-13, Map1, Map1+1, Map1-6, VirB10, VirB9, Q5FFE6, Q5HAR6, Q5HBI2, Q5HA95; **Band 11**: Map1, Map2, BamA, DnaK, GroEL, FusA, Def, 50S-L4, PyrE, X5HG56, Q5HBI2; **Band 12**: 30S-S18, 30S-S12, 50S-L7/L12, 50S-L18, 50S-L24, 50S-L28 X5HG56, Q5HBN6; **Band 13**: HupB, X5HG56; **Band 14**: 30S-S12, 50S-L7/L12, 50S-L18, GroEL, YajC, PyrE.

The first step in this study was to recover most of the OM complex with minimal contamination by cytoplasmic and inner membrane fractions. To do this, we used sarkosyl, an ionic detergent commonly used in the purification of OMs in Gram-negative bacteria, because it selectively solubilizes cytoplasmic and inner membranes while conserving the integrity of the OM [24]. Fig. 1 shows the workflow used to obtain the OM fraction. To assess protocol efficacy, samples were harvested at critical time points during the purification process, and their quality was evaluated using TEM, SDS-PAGE to identify proteins in the most intense bands, and Western blotting (Fig. 2). After sarkosyl treatment of intact EBs (Fig. 2A), empty shells with spherical morphology, corresponding to the OM fraction, were observed (Fig. 2B). These OM complexes, with a diameter of approximately 200 nm, appeared to be devoid of inner membrane and cytoplasm components, in contrast to intact EBs (Fig. 2A). Comparative protein migration profiles of the different fractions (intact EBs, E; sarkosyl soluble fractions, S; and outer membrane fractions, OMs) were analyzed using SDS-PAGE (Fig. 2C), and each subcellular fraction displayed a distinct migration pattern. The OM preparation showed prominent bands at approximately 134, 63, 55, 41, 37, and 29 kDa. The most abundant proteins, in the 30 kDa range, may represent Map1 protein family. When the different fractions were analyzed via Western blot using a monoclonal antibody against Map1 (a specific OM marker), intact EBs (the positive control) displayed a strong ~30 kDa band corresponding to Map1 (Fig. 2D). This protein was detected in the OM fraction but not in the soluble fraction, confirming the efficacy of the purification protocol (Fig. 2D). Altogether, these results clearly indicate that the insoluble sarkosyl fraction was strongly enriched with *E*. *ruminantium* OM complexes.

### *In silico* subcellular localization prediction of *E*. *ruminantium* proteins

We utilized a combination of three computational prediction tools, CELLO 2.5, PSORTb 3.0, and MetaLocGramN, to predict subcellular localization in the entire *E*. *ruminantium* proteome. These programs have been used to identify OMPs in several Gram-negative bacterial species [35–37]. Though the programs made diverse subcellular localization predictions for the same proteins, the combination of different predictors minimizes the risk of false positives for OMP prediction. PSORTb 3.0, CELLO 2.5, and MetaLocGramN predicted 490, 461, and 526 cytoplasmic proteins in *E*. *ruminantium* (~50% of total proteins), respectively (Table 1). CELLO 2.5 predicted 11.5% of proteins were inner membrane proteins (IMPs), whereas the two other programs predicted roughly twice as many (20%). CELLO 2.5 identified the highest proportion of OMPs (9.4%, 90/948), followed by MetaLocGramN (7.4%, 71/948) and PSORTb 3.0 (1.1%, 11/948). PSORTb 3.0 could not predict the localization of 236 proteins, while CELLO could not provide predictions for 256.

**Fig 3:**
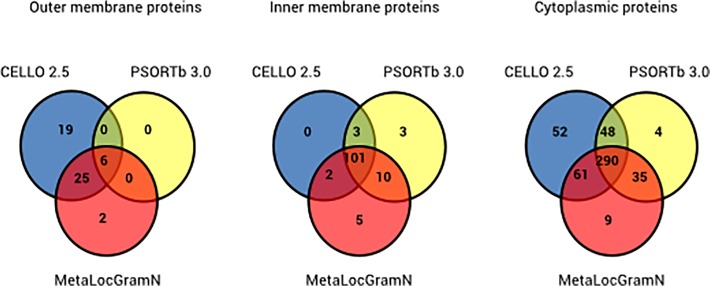
Venn diagram representing the predicted subcellular localization of *E*. *ruminantium* proteins using PSORTb 3.0, CELLO 2.5, and MetaLocGramN. The data presented result from consensus prediction of subcellular localization.

Altogether, we predicted that the total proteome of *E*. *ruminantium* (948 proteins) consisted of 53% (499/948) cytoplasmic proteins, 13% (124/948) IMPs, and 5.4% (52/948) OMPs (Table 1). In Fig. 3, the number of proteins in each Venn diagram compartment corresponds the consensus prediction correctly predicted by an algorithm for a given subcellular localization. Of the 52 OMPs identified using consensus predictions, 6 were identified by all three programs. Twenty-one were predicted by only a single program: 19 for CELLO 2.5 and 2 for MetaLocGramN. CELLO 2.5 predicted the highest number of consensus OMPs (50), followed by MetaLocGramN (33) and PSORTb 3.0 (6). All three programs identified two hundred and ninety cytoplasmic proteins. CELLO 2.5 predicted the highest number of cytoplasmic proteins, whereas PSORTb 3.0 predicted the lowest.

### Identification of proteins in the *E*. *ruminantium* OM fraction

OM fractions prepared from three biological replicates were analyzed individually using 1DE-nanoLC-MALDI-TOF/TOF MS. The proteins identified are presented in Table 2. Of the 46 non-redundant proteins identified in the OM fraction, 41 had known functions (either characterized experimentally or annotated via high sequence similarity), and the remaining five proteins were classified as hypothetical proteins. Several of these proteins (e.g. ERGA_CDS_04510, ERGA_CDS_04580) are conserved among members of *Anaplasmataceae*. Of the 46 proteins identified, 39% were indeed OMPs (18/46), 11% were IMPs (5/46), and 50% (23/46) were cytoplasmic. These proteins were classified into four functional groups: structural and transport proteins, biogenesis proteins (e.g. BamA, ERGA_CDS_08660), virulence proteins, and proteins involved in metabolic processes (e.g. GroEL, ERGA_CDS_06640 and Ef-Tu, ERGA_CDS_01580). Several ribosomal proteins and chaperones were also identified. Of the 18 OMPs identified, 5 belonged to the well-known MAP1 family (Map1, Map1+1, Map1-6, Map1-13, and Map1-14), 2 comprised β-barrel assembly machinery (BamA and BamD), 3 were components of the type IV secretion system (VirB9-1, VirB9-2, and VirB10), 1 was a porin, and 1 was a major ferric iron-binding protein. The six putative uncharacterized proteins had neither functional annotations in UniProt, nor hits in the Pfam database. Two of these (ERGA_CDS_04580, ERGA_CDS_05150) were predicted by SignalP to contain signal peptides. The first had no homology with known proteins and seemed to be unique in the *E*. *ruminantium* genome, whereas the second had similarity to ECH_0525, an ortholog of Esp73, an OMP in *Anaplasma phagocytophilum*.

**Table 2:** Proteins identified in the outer membrane fraction of *E*. *ruminantium* via 1DE-nanoLC-MALDI-TOF/TOF.

Locus tag	Protein	Acession Number	Function	Protein MM(kDa)	Number of peptides^a^	Protein score^b^	Coverage (%)	PSORTb 3.0	CELLO 2.5		MetaLocGramN		Consensus prediction
**Outer membrane proteins (39%)**													
ERGA_CDS_00150	VirB10	Q5HCE9	Virulence	48.717	2	294	9	I	E	P	C	C	OM
ERGA_CDS_00160	VirB9–2	Q5HCE8	Virulence	30.993	2	86	6	C	OM			C	OM
ERGA_CDS_01230	Possible major ferric iron binding protein	Q5FFA9	Transport/virulence	41.309	2	188	14	OM	C	OM		I	OM
ERGA_CDS_02370	Hypothetical protein	Q5FFH4	Unknown	37.402	3	259	26	U	OM			E	OM
ERGA_CDS_02510*	Hypothetical protein	Q5HBS6	Unknown	90.496	1	26	1	U	OM			OM	OM
ERGA_CDS_03960*	Hypothetical protein	Q5HBE1	Unknown	55.237	1	23	2	U	OM			E	OM
ERGA_CDS_04510	Hypothetical protein	Q5FGV5	Unknown	134.574	1	124	2	U	C	OM		OM	OM
ERGA_CDS_04580	Putative exported protein	Q5HB83	Porin	41.826	9	832	47	U	OM			OM	OM
ERGA_CDS_05150^#^	Putative exported protein	Q5FH07	Unknown	63.139	18	827	27	OM	OM			OM	OM
ERGA_CDS_07300	Hypothetical outer membrane protein	Q93FS2	Cell struture	28.127	2	107	18	U	C			E	OM
ERGA_CDS_07840	VirB9–1	Q5HAC9	Virulence	29.489	2	153	10	I	C	OM		C	OM
ERGA_CDS_08100	Putative exported lipoprotein	Q5HAA5	Outer membrane assembly	29.344	1	119	10	OM	C	OM		OM	OM
ERGA_CDS_08660	Outer membrane protein omp1	Q5FGI9	Outer membrane assembly	87.257	2	173	5	OM	OM			OM	OM
ERGA_CDS_09000	Map1-13	Q4L0D3	Cell struture	32.965	4	419	28	I	OM	P		C	OM
ERGA_CDS_09010	Map1-14	Q4W4X7	Cell struture	34.186	1	122	7	U	OM			OM	OM
ERGA_CDS_09090	Map1-6	Q4L0C5	Cell struture	33.736	4	529	36	OM	OM			OM	OM
ERGA_CDS_09160	Map1	Q46330	Cell structure	31.204	5	948	38	OM	E			E	OM
ERGA_CDS_09170	Map1+1	Q4L0B8	Cell structure	31.817	3	136	14	U	OM			OM	OM
**Inner membrane proteins (11%)**													
ERGA_CDS_01470	Major antigenic protein 2 SCO2 like-protein	Q9R416	Cell struture	23.562	1	79	10	I	P	C		I	I
ERGA_CDS_03170	Phosphatidylserine decarboxylase proenzyme	Q5FHJ9	General metabolism	25.245	1	103	7	I	I			I	I
ERGA_CDS_05400	VirB4	Q5FFK8	Virulence	90.865	1	82	2	I	C			C	I
ERGA_CDS_06350	Putative Protease IV	Q5HAR6	General metabolism	32.263	2	157	14	I	C	OM		I	I
ERGA_CDS_08130	Preprotein translocase. YajC subunit	X5HHA7	Cellular processes and signaling	13	1	43	15	I	C	P		I	I
**Cytoplasmic proteins (50%)**													
ERGA_CDS_01570	Elongation factor G	Q5FFE7	Protein synthesis	76.042	1	40	3	C	C			C	C
ERGA_CDS_01580	Elongation factor Tu	Q5FFE6	Protein synthesis	43.282	5	344	17	C	C			C	C
ERGA_CDS_01760	Peptide deformylase	Q5HBZ5	Cell process	21.926	1	43	9	C	C			C	C
ERGA_CDS_02930	Putative DNA-binding protein HU-beta	Q5HBN6	Cell process	10.665	1	94	19	U	C	P		E	C
ERGA_CDS_03000^#^	Helix-turn-helix domain protein	X5HG56	DNA binding	12250	1	40	6	U	C			E	C
ERGA_CDS_03230	Response regulator pleD	Q5HBK9	Regulation	52.358	1	154	5	C	C			C	C
ERGA_CDS_03510	Putative peroxiredoxin	Q5HBI2	General metabolism	23.349	8	760	52	C	C			C	C
ERGA_CDS_03570	Polyribonucleotide nucleotidyltransferase	Q5FHK5	General metabolism	86.507	1	77	3	C	OM			C	C
ERGA_CDS_07810	Inosine-5'-monophosphate dehydrogenase	Q5FGA7	General metabolism	52.348	1	106	6	C	C			C	C
ERGA_CDS_08210	Putative response regulator	Q5HA95	Regulation	30.477	2	118	9	C	C			C	C
ERGA_CDS_08900	Orotate phosphoribosyltransferase	Q5FGJ6	General metabolism	22.343	1	57	7	C	C			C	C
ERGA_CDS_09220	Cell division protein FtsZ	A1XRC7	Cell division	46.126	1	58	4	I	C	E		I	C
***Chaperones***													
ERGA_CDS_02450	Chaperone protein HtpG	Q5FHC4	Chaperone	72485	1	60	3	C	C			C	C
ERGA_CDS_05670	Chaperone protein DnaK	Q5FFM4	Chaperone	69. 957	2	183	6	C	C			C	C
ERGA_CDS_06640	Chaperonin. 60 kDA (GroEL) protein	Q5FFZ1	Chaperonin	58. 859	16	1473	43	C	C			C	C
***Ribosomal proteins***													
ERGA_CDS_01550	30S ribosomal protein S12	Q5FFE9	Translation	13.671	1	49	23	C	P	C		C	C
ERGA_CDS_01640	50S ribosomal protein L7/L12	Q5HC06	Translation	14.269	1	59	15	C	C			C	C
ERGA_CDS_05500	50S ribosomal protein L28	Q5FFP1	Translation	11.53	1	63	18	C	C			C	C
ERGA_CDS_06130	50S ribosomal protein L18	Q5FFR4	Translation	14.051	1	39	11	C	C			C	C
ERGA_CDS_06150	30S ribosomal protein S8	Q5FFV4	Translation	14.668	1	105	11	C	C	OM	P	C	C
ERGA_CDS_06180	50S ribosomal protein L24	Q5FFV1	Translation	11.7	1	57	28	C	P	C	E	C	C
ERGA_CDS_06280	50S ribosomal protein L4	Q5FFU1	Translation	23.28	1	55	9	C	C	P		C	C
ERGA_CDS_06340*	30S ribosomal protein S1	Q5HAR7	Translation	63.425	1	22	2	C	OM			C	C

Their predicted subcellular localization is shown by U, unknown; C, cytoplasmic; I, inner membrane; O, outer membrane; E, extracellular; P, periplasmic.

^a^Number of unique peptides that match the sequence of the identified protein

^b^MASCOT Score—Identified proteins were only considered if a protein score above 40 was obtained (p<0.05)

^#^Hypothetical/uncharacterized proteins that had a significant hit on the BLASTp searches. The name of the BLASTp search best hit is here presented.

* proteins identified below the Mascot score (>20) and considered for the study as peptides were checked and interpreted manually to confirm the MASCOT suggestion

In summary, our study increased the number of OMPs experimentally identified accounting for 34% of total predicted OMPs in *E*. *ruminantium* (18/52), whereas the total number OMPs account only for 5.5% of *E*. *ruminantium* proteome (52/948). Thus, the OM purification process described enriched OMPs.

## Discussion

The OM of Gram-negative bacteria is an important interface between the outside and inside of the cell. It protects bacteria against hostile environments. OMPs fulfill a number of crucial functions, such as supporting the biogenesis and integrity of the OM and acting as porins and virulence factors, playing a fundamental role in adherence to host cells, invasion, and evasion of host-defense mechanisms [38].

The purification of OMs is a key step in the identification of OMPs. Several methods, such as isopycnic centrifugation using a sucrose gradient, addition of Triton X-100, and carbonate extraction protocols, have been tested in bacteria [9–11,39]. However, the sarkosyl solubilization strategy, which solubilizes IM proteins and separates IM and OM proteins [24], has become the preferred method for many Gram-negative bacteria, due to the higher purity and better reproducibility of the OM extracts obtained in this manner [13,40,41]. By applying this method to *E*. *ruminantium* EBs, we obtained a highly enriched OM fraction. Our proteomic analysis led to the identification of 18 unique OMPs corresponding to 34% of total cell OMPs. The low percentage of sarkosyl-insoluble proteins obtained may be due to excessive washing of the pellets after sarkosyl treatment, resulting in loss of proteins or lysis of cells [10,25]. In addition, OMP extraction was performed on the extracellular, infectious form of *Ehrlichia*. It is likely that only certain *E*. *ruminantium* proteins are expressed at a given life cycle stage [42]. For instance, expression of most *E*. *chaffeensis* proteins varies depending on host and vector environments and stage of development [43,44].

We also analyzed the entire *E*. *ruminantium* proteome to determine the theoretical subcellular localization of all proteins (OM, IM, cytoplasmic, periplasmic, or extracellular). These *in silico* predictions allowed us to estimate the quality of the enrichment of OMPs in the OM fraction obtained using our purification protocol. PSORTb 3.0 is one of the most precise subcellular localization predictor for many Gram-negative bacteria [32]. It uses a combination of factors based on motif and profile analyses, e.g. the presence of signal peptides, OM motifs, transmembrane helices, and similarity to proteins with known localization [32]. However, in this study, it returned a high number of proteins with unknown localization (236 or 24.8% of total proteins). This problem may be due to the absence of significant sequence similarity between some *E*. *ruminantium* proteins and proteins in the PSORTb 3.0 database. Similar results have been observed in numerous other bacteria [34]. Consequently, we chose two other computational localization predictors to overcome this weakness. CELLO 2.5 has the advantage of using multiple Support Vector Machines (SVMs) to analyze four types of protein descriptors, including amino acid composition, dipeptide composition, partitioned amino acid composition, and frequency of residues with particular physicochemical properties [33], yielding better predictive performance [33]. However, in our study, CELLO 2.5 predicted multiple localization sites for 256 proteins that were subsequently grouped in a “unknown localization” category [35]. Finally, we included MetaLocGramN program, a meta-predictor that combines multiple primary methods, including general subcellular localization, signal peptide predictors, transmembrane helix predictors, and beta barrel OMP predictors [34]. The combination of results from these three programs improved the accuracy of subcellular localization predictions [9,35,45].

Collectively, our bioinformatics analysis predicts that 5.4% of the annotated genes in the *E*. *ruminantium* genome are OMPs. Analyses of other Gram-negative bacteria have identified approximately the same percentage of predicted OMPs. For example, an analysis employing 10 different predictors to analyze the *Pasteurella multocida* genome identified 98 OMPs in an avian strain and 107 in a porcine strain (4.8% and 5.0% of total proteins, respectively) [46]. Similarly, prediction of the subcellular localization of *P*. *syringae* Lz4W proteins, performed using PSORTb 3.0, revealed that 148 out of a total of 1,479 proteins (10%) were OMPs [11]. In addition, we compared our results to those obtained experimentally from many other bacteria. In *L*. *pneumophila*, OM and surface-exposed proteome analyses using cellular fractionation and fluorescent labeling led to the identification of OMPs accounting for 8.5% of total proteins [12]. These results suggest that our prediction of *E*. *ruminantium* OMPs yielded a reasonable identification rate.

We experimentally identified a total of 46 non-redundant proteins in the OM fraction, 18 of which were clearly classified as OMPs. These 18 OMPs correspond to 1.9% of the entire *E*. *ruminantium* proteome (18/948) and 34.6% of predicted OMPs in the entire proteome (18/52). Previous studies on the total *E*. *ruminantium* proteome have identified 64 non-redundant proteins including 8 OMPs [17]. Thus, as expected, enriching the OM fraction resulted in an increased number of OMPs being identified. Some of these OMPs have known functions and include proteins of the Map1 cluster [47], BamA/D [48], VirB9-1 [49], VirB9-2, VirB10 [50], a porin [51], and major ferric iron-binding protein [52]. We also characterized five proteins classified as hypothetical but predicted to be OMPs, including ERGA_CDS_04510, 03960, 02510, 02370, and 05150. BLAST search on ERGA_CDS_05150 revealed an ortholog in *Ehrlichia chaffeensis*, Esp73; an ortholog to *A*. *phagocytophilum* Asp55 and Asp62, that is predicted to contain 22 transmembrane β-strands forming a β-barrel and, thus, may be involved in membrane transport [53]. Further functional characterization of these newly discovered OMPs should be carried out to evaluate their potential as protective antigens.

Map1, the immunodominant, major OMP expressed by *E*. *ruminantium* in the mammalian host, is encoded by a member of a multigene family comprising 16 paralogs [54]. The number of Map1 family proteins detected in this study (n = 5: Map1, Map1+1, Map1-6, Map1-14, and Map1-13) was greater than that detected in a previous proteomic analysis [17]. These proteins are known to be differentially transcribed *in vitro* in endothelial and tick cell cultures [54,55] and are well conserved, since *omp-1*, *msp2*, *p44*, *p30*, and *map-1* belong to a superfamily harboring the PF01617 Pfam domain [1]. Map1 family proteins are considered priority targets for candidate vaccines [56], as they are potentially involved in *E*. *ruminantium* adaptation to the mammalian host and its vector, the tick [18]. However, few data are currently available on the expression and characterization of Map1 family proteins throughout the bacterial life cycle [17].

Proteins of the β-barrel Assembly Machinery (BAM) complex are involved in diverse cellular functions, including solute transport, protein secretion, and assembly of protein and lipid components of the OM [57]. They account for the vast majority of bacterial OMPs and are essential for bacterial viability and function [58]. The insertion of proteins in the OM depends on a protein complex that contains the OMP BamA and four associated lipoproteins (BamB, C, D, and E) [59]. BamA (ERGA_CDS_08660) and BamD (ERGA_CDS_08100) were identified in our experimental analysis. BamA proteins are essential for the biogenesis of β-barrel OMPs and play a central part in OMP assembly [60–62]. It has been observed that reducing the levels of BamA significantly affects the ability of the β-barrel membrane protein OprF to localize to the OM, showing its essential role in OM biogenesis [61]. BamD is the only essential lipoprotein in the BAM complex [63], and it is highly conserved in Gram-negative bacteria as well [64].

Many bacterial species use specialized secretion systems to transfer macromolecules across membranes [65]. The type IV secretion system (T4SS) translocates DNA or proteins across membranes directly into eukaryotic host cells to subvert host cellular functions. Consequently, the proteins that make up this system represent crucial bacterial virulence determinants in important human pathogens such as *B*. *henselae*, *Helicobacter pylori*, *L*. *pneumophila*, *Bordetella pertussis*, and *Brucella melitensis* [66,67]. In this study, we identified three conserved pathogenesis-associated proteins: VirB4, VirB9, and VirB10. VirB9 is an OM component of the T4SS and is hypothesized to be a translocation pore [68,69]. It is essential for the stability of the translocation machinery and substrate selection [69]. It interacts with VirB10, which bridges the IM and OM protein subcomplexes, and actively participates in T4SS substrate transfer across the bacterial envelope [12,70–72]. VirB4 is an ATPase, providing energy for substrate export and pilus biogenesis, and it interacts with several other VirB proteins, such as VirB10 [50]. It is not surprising, then, to identify such proteins in the *E*. *ruminantium* OM fraction. Moreover, a recent study showed that some T4SS components could be potential vaccine candidate for pathogenic bacteria [49].

We also identified a porin (ERGA_CDS_04580) that has no homology to other proteins and that seems to be unique to *E*. *ruminantium*. Porins play a fundamental role in pathogenicity [51], participating in adhesion to and invasion of host cells and evasion of host defense mechanisms [73]. They represent good targets for therapeutic development. Some porins activate immunological responses, induce signaling pathways, and modify the properties of the OM lipid barrier [73]. It would be interesting to further investigate the role of this porin with functional studies.

The periplasmic major ferric iron binding protein of Gram-negative bacteria (ERGA_CDS_01230), which has homologous counterparts in many other pathogenic species, plays a key role in the acquisition of iron from mammalian host serum iron transport proteins; thus, it is essential for the survival of the pathogen within the host [40,74].

Within the cell, the full-length protease (ERGA_CDS_06350), may be processed into the intermediate 45 kDa form, which represents a form of protease IV that lacks the signal sequence. This 45 kDa intermediate may undergo a conformational change that activates its protease activity, triggering the cleavage of the propeptide from the mature protease domain. The mature protease IV may be secreted through the OM, functioning in the developmental cycle [75,76] and as an important virulence factor [77].

In this study, we detected the chaperones DnaK and GroEL in the OM fraction, though they are depicted as cytoplasmic proteins. These results are not surprising, as these proteins are often membrane-associated [13,78]. In many bacteria, such as *L*. *pneumophila* and *Borrelia burgdorferi* [12,79], GroEL (Hsp60) is found in the OM and plays a role in the folding of a large number of proteins; in other bacteria, this protein is active in bacterial adhesion [80,81]. Similarly, in *E*. *chaffeensis*, the chaperone proteins GroEL and DnaK, and the translation elongation factor G, are localized to the membrane surface [82]. GroEL has also been detected on the surfaces of *H*. *pylori* [83], *L*. *pneumophila* [84], *Haemophilus ducreyi* [85], and *Clostridium difficile* [80] via immunofluorescence or immunoelectron microscopy. Finally, DnaK has been detected on the surface of *H*. *pylori* [83]. Other important cytoplasmic proteins identified in our study (FusA, TypA, EF-Tu, and Tig) are associated with ribosomes but can be membrane-associated during the transport of nascent OMPs across the periplasmic space to the OM [86]. Recently, EF-Tu was shown to be membrane-associated, secreted in outer membrane vesicles (OMVs), and immunogenic during *Burkholderia* infection in a murine model of melioidosis [87]. Therefore, we cannot deny the possibility that these proteins with well-known functions in the cytoplasmic, periplasmic, or inner membrane are present in the OM of *E*. *ruminantium* and play unexpected roles in *E*. *ruminantium* -host interaction.

Surprisingly, we also detected ribosomal proteins with a predicted cytoplasmic localization. These proteins may represent a contamination with cytoplasmic proteins. Such proteins have also been identified in OM fractions of *Pseudomonas* and *Yersinia* strains, however [88,89]. Moreover, it should be noted that among these ribosomal proteins, we obtained a majority of 50S ribosomal subunits, as has been shown in *Legionella* [12]. Interestingly, one ribosomal protein we found in the OM fraction (ERGA_CDS_01640) has been predicted by S4TE software as a putative type IV effector [27]. Type IV effectors are proteins produced by pathogenic bacteria to manipulate host cell gene expression and other processes and have been shown to be critical for pathogenicity, making them salient targets for understanding bacterial virulence [90]. The function of this particular protein and its role in *E*. *ruminantium* pathogenicity is currently under investigation.

## Conclusion

This study provides the first proteomic profile of the *Ehrlichia ruminantium* OM. The combination of subcellular fractionation via sarkosyl solubilization and a high degree of accuracy in predicting OMP status allowed us to generate a high-resolution OM proteome comprised of 46 proteins identified in the OM fraction. We identified OMPs involved in cell wall structure, i.e. at the interface between bacteria and host cells, and proteins known to be virulence factors. Moreover, we identified new OMPs by our approach coupling a consensus of computer algorithms, manual sequence analysis and experimental proteomics. In the future, functional studies should explore the potential of using these OMPs as vaccine candidates against *E*. *ruminantium*.

## Supporting Information

S1 Table Proteins subcellular localization prediction from Ehrlichia ruminantium (strain Gardel) genome. 948 proteins were analyzed using 3 bioinformatic predictors and the resulting consensus prediction is indicated.
(XLSX)
